# Acute promyelocytic leukemia in super male

**DOI:** 10.1002/jha2.518

**Published:** 2022-08-02

**Authors:** Reshma Roshan, Sajad Ahmed Geelani, Santosh Govind Rathod, Afaq Ahmad Khan, Aakash Chozakade, Ayeshah Jalid, Javid Rasool Bhat

**Affiliations:** ^1^ Department of Clinical Hematology SKIMS Srinagar India

Dear Editor,

A 13‐year young male initially presented in hematology department with generalized weakness, bleeding, and ecchymosis over the abdomen. Laboratory evaluation showed the following parameters: white blood cells (WBC) 12 × 10^9^/L, hemoglobin (Hb) 6.3 g/L, mean corpuscular volume 87 fL, and platelets 7 × 10^9^/L. The results of a coagulation profile were as follows: prothrombin time 18.1 s (reference: 11–14.5 s); fibrinogen 1.16 g/L (reference: 2–4 g/L); active partial thromboplastin time 42.1 s (reference: 28–40 s); thrombin time 18.1 s (reference: 14–21 s); d‐dimer 8688 mg/ml (reference: 0–0.5 mg/ml). Peripheral blood smear examination showed promyelocytes 97%, neutrophils 2%, and lymphocytes 1% (Figure 1, panel A). Bone marrow aspiration shows promyelocytes were of uneven cell size with irregular nuclear shapes, such as folded, segmented, and binucleate. Cytogenetic examination revealed translocation of chromosome (15;17) with super male karyotype (Figure 1, panel B). Promyelocytic leukemia‐retinoic acid receptor alfa (PML‐RARA) by PCR was positive with qPCR showing 68.8% transcript. The patient started on all‐trans‐retinoic acid at 45 mg/m^2^ and arsenic trioxide at 0.15 mg/kg as induction and doing well. XYY syndrome is a rare sex chromosome aneuploidy, with an incidence of one in 1000 males. The phenotypic spectrum includes tall stature, learning disabilities, aggressive behavior, and normal sexual development with normal testosterone levels. The patient had tall stature and aggressive behavior, with normal sexual development and testosterone level. Postinduction bone marrow was in morphological remission with no leukemic promyelocyte, and PML‐RARA by qPCR showed 0.016% transcript. In the present case, XYY cytogenetics was germline and was not associated with leukemic clones that are Y+. Repeat cytogenetics after induction showed XYY karyotype. Association between acute promyelocytic leukemia and supermale karyotype is rare and patients are more prone to hematological malignancy. Acute promyelocytic leukemia can present with additional chromosomal abnormality like XYY syndrome. A patient needs close follow‐up in the future.

**FIGURE 1:**
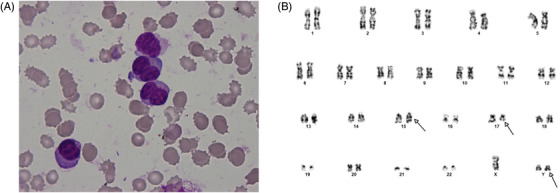
(A) Promyelocytes were of uneven cell size with folded nuclear shape. (B) Cytogenetic examination revealed translocation of chromosome (15;17) with XYY karyotype

## CONFLICT OF INTEREST

The authors declare they have no conflicts of interest.

## FUNDING INFORMATION

The authors received no specific funding for this work.

## ETHICAL APPROVAL

Institutional ethical committee approval was taken for this study.

## AUTHOR CONTRIBUTIONS

RW, SG, SR, and AK treated the patients, designed the study, and analyzed the data. AC, AJ, and JB wrote the manuscript and did a review of the literature. And all authors checked and approved the manuscript.

## INFORMED CONSENT

Informed consent was obtained from the patient.

## Data Availability

Data can be made available as per request by the corresponding author of the article.

